# Adolescent idiopathic scoliosis: surgical treatment and quality of life

**DOI:** 10.1590/1413-785220172503157788

**Published:** 2017

**Authors:** Luciano Miller Reis Rodrigues, Alberto Ofenhejm Gotfryd, André Nunes Machado, Matheus Defino, Leonardo Yukio Jorge Asano

**Affiliations:** 1 Faculdade de Medicina do ABC, Santo André, SP, Brazil; 2 Santa Casa de Misericórdia de São Paulo, Departamento de Ortopedia e Traumatologia, "Pavilhão Fernandinho Simonsen", SP, Brazil

**Keywords:** Scoliosis, Adolescent, Spinal fusion, Pedicle screws, Treatment outcome

## Abstract

**OBJECTIVES:**

The purpose of this study was to determine the influence of perioperative factors and their impact on clinical and functional outcomes in Brazilian patients with adolescent idiopathic scoliosis (AIS).

**METHODS:**

We performed a prospective study with 49 consecutive AIS patients who underwent spine fusion and had a minimum 2 year follow-up. Clinical and radiographic data were correlated to SRS-30 scores in order to predict postoperative results.

**RESULTS:**

There was a negative association between patient age at the time of surgery and back pain. We also observed higher scores in the "satisfaction" domain in patients who underwent surgery after 15 years of age (p < 0.05). The average SRS-30 "mental health" score was significantly higher in males than in females (p= 0.035). Patients treated with braces had worse results than those who did not use them (p= 0.005).

**CONCLUSIONS:**

Posterior spine fusion led to improvement of all domains of the SRS-30 questionnaire. Clinical results were influenced by age, sex and the use of braces prior to surgery. There was no correlation between curve correction and presence of perioperative complications. ***Level of Evidence IV, Case Series.***

## RESUMO

**OBJETIVO:**

A finalidade deste estudo foi determinar a influência dos fatores perioperatórios e seu impacto sobre os desfechos clínicos e funcionais em pacientes brasileiros com escoliose idiopática do adolescente (EIA).

**MÉTODOS:**

Foi realizado um estudo prospectivo com 49 pacientes consecutivos com EIA submetidos *à* fusão da coluna vertebral, com seguimento de no mínimo dois anos. Os dados clínicos e radiográficos foram correlacionados com o escore SRS-30 para predizer os resultados pós-operatórios.

**RESULTADOS:**

Houve uma associação negativa entre a idade do paciente no momento da cirurgia e dor nas costas. Observamos também escore mais alto no domínio "satisfação" nos pacientes operados depois dos 15 anos de idade (p < 0,05). O escore médio de "saúde mental" do SRS-30 foi significativamente superior em homens com relação às mulheres (p= 0,035). Os pacientes tratados com *órteses* tiveram resultados piores comparados com aqueles que não usaram (p= 0,005).

**CONCLUSÃO:**

Artrodese posterior levou *à* melhora de todos os domínios do questionário SRS-30. Os resultados clínicos foram influenciados por idade, sexo e uso de *órtese* antes da cirurgia. Não houve correlação entre a correção da curva e a presença de complicações perioperatórias. ***Nível de Evidência IV, Série de Casos.***

## INTRODUCTION

Adolescent idiopathic scoliosis (AIS) is a three-dimensional disease corresponding to 70 to 80% of all spine deformity cases. The prevalence of AIS is described as 2-3%; 0.3 to 0.5% of these cases are progressive and consequently require surgical treatment.^1^

The main goals of AIS surgery are to obtain a balanced trunk and solid fusion. However, despite good clinical and radiological outcomes patient self-evaluation and quality of life may be poor after surgery. Several aspects influence postoperative outcomes for AIS, including age, ethnicity, sociocultural issues and sex. Identification of these variables before surgery procedures may therefore help predict treatment outcomes.^2^ ^-^ ^5^ The authors of this present study did not find previous studies evaluating these parameters in Brazilian AIS patients in the medical literature.

The objective of this study was to determine the influence of perioperative factors and their impact on clinical and functional outcomes in Brazilian AIS patients.

## PATIENTS AND METHODS

We performed a prospective study with forty-nine consecutive AIS patients who underwent spine fusion surgery between March 2009 and June 2011. There was no restriction to curve patterns and all AIS types according to Lenke's classification were included.^6^ The same senior surgeon performed all procedures. Evaluations were performed preoperatively and 6, 12 and 24 months after the surgery. Approval was obtained from the institutional review board of our institution (process 377.252) and all patients voluntarily signed a informed consent form. 

### Inclusion criteria

We included AIS patients with Cobb angle >45°, age between 11 and 18 years at the time of surgery, instrumentation with pedicle screw only and minimum of two years follow-up. Patients who had no follow-up or incomplete data were excluded.

High thoracic rib hump during Adam's forward bending test and coronal translation of the trunk (evaluated with a plumb line) were measured as clinical parameters. Coronal deviations greater than 20 mm were considered to be trunk imbalance.^7^

Function was evaluated using the SRS-30 questionnaire developed by the Scoliosis Research Society. It consists of 30 questions divided into five domains (pain, function, appearance, mental health and satisfaction). Each question ranges from 1 (worst scenario) to 5 (best scenario) and the maximum score is 150. In the present study we used a culturally adapted and validated questionnaire in Brazilian Portuguese.^8^ A trained nurse who was not directly involved in the study applied all the questionnaires.

The following parameters were correlated with the SRS-30 results: (1) age at time of surgery; (2) use of braces before surgery; (3) main thoracic Cobb angle; (4) main thoracic curve correction; (5) sex; and (6) complications.

The correction percentages of the main thoracic curve were established using the equation proposed by Cheung:^9^

% Correction = Preoperative Cobb angle - Postoperative Cobb angle x 100% Preoperative Cobb angle

Radiographic assessments comprised posteroanterior and lateral spine x-rays in a standing position. In all cases the Cobb angle of the proximal thoracic curve and main thoracic and thoracolumbar/lumbar curves were measured.^5^ The curves were classified according to Lenke's criteria.^6^ Curve flexibility was assessed by means of supine lateral bending x-rays.^7^ ^,^ ^10^

### Statistical analysis

The sample was characterized by frequency and percentage for categorical variables and by mean, standard deviation (SD), median, minimum and maximum values and number of valid observations for numeric variables. Domain scores for the SRS-30 questionnaire were described in each group and time by mean, SD, median, minimum and maximum values and number of valid observations.

The relationship between age and domain scores for the questionnaire in each moment was verified by Pearson's correlation. To compare questionnaire scores between groups and across time, we used analysis of variance (ANOVA) with repeated measures and a fixed factor, considering that normality and/or homogeneity of variances for variables can be assumed between each group of interest. In each ANOVA the interaction effect between time and group was tested to verify the need to compare the groups in each stage separately, in other words, to check whether the behavior of groups through time follows the same trend.

When statistical significance was observed in the coefficient test between age and domain scores of the questionnaire, linear regression analysis was used to interpret the regression coefficient. A significance level of 95% (α= 0.05) was used to complete each test (bilateral); in other words, descriptive levels (p) of less than 0.050 were considered statistically significant. SPSS software version 19.0 was used.

## RESULTS

Of the 63 patients with AIS who underwent surgical correction, 49 patients (78%) completed the SRS-30 questionnaire before surgery and 2 years after surgery. Mean participant age (±SD) at the time of surgery was 11.9 (±1.2 years), ranging from 11 to 18 years and 87.8% of them were female. Prior to surgery, 67% of patients did not use braces. The most common curve patterns were Lenke 1AN (46.9%) and 1BN (24.5%). Most patients were considered to have "coronal imbalance" before surgery (61.2%). Of all patients, 81% were classified as grade 4 or 5 Risser sign. (Table 1)

**Table 1:** Sample features.

Age (years)		
Mean ± SD.	11.9 ± 1.2	
Median		11.0
Minimum - Maximum	11 - 18	
Total of patients		49
Sex - n (%)		
Male	6	(12.2%)
Female	43	(87.8%)
Total of patients	49	
Brace - n (%)		
No	33	(67.3%)
Yes	16	(32.7%)
Total of patients	49	
Lenke - n (%)		
1AN	23	(46.9%)
1A-	4	(8.2%)
1B+	1	(2.0%)
1BN	12	(24.5%)
1B-	2	(4.1%)
1CN	2	(4.1%)
2	2	(4.1%)
3	2	(4.1%)
5	1	(2.0%)
Total of patients	49	
Plumb line - n (%)		
Compensated	30	(61.2%)
Uncompensated	19	(38.8%)
Total of patients	49	
Risser sign - n (%)		
0, 1, 2, 3	9	(18.4%)
4, 5	40	(81.6%)
Total of patients	49	
Proximal thoracic angle		
Mean ± SD.	25.4 ± 9.4	
Median		25.0
Minimum - Maximum	8 - 56	
Total of patients		49
Main thoracic angle		
Mean ± SD.	58.5 ± 11.8	
Median		58.0
Minimum - Maximum	20 - 91	
Total of patients		49
Thoracolumbar angle		
Mean ± SD.	36 ± 10.1	
Median		37.0
Minimum - Maximum	17 - 56	
Total of patients		49
Rib hump size		
Mean ± SD.	2.2 ± 0.9	
Median		2.0
Minimum - Maximum	0 - 4	
Total of patients		49

The mean proximal thoracic Cobb angle was 25.4 (±9.4). The mean main thoracic and thoracolumbar/lumbar Cobb angles were 58.5 (±11.8) and 36.0 (±10.1), respectively. Mean size of the thoracic hump was 2.2 cm (±0.9 cm). The average correction of the main thoracic and thoracolumbar/lumbar Cobb angles was 39.8.

A negative association between patient age at the time of surgery and back pain was found. Patients who underwent surgery after 15 years of age had worse outcomes compared to those who had surgery before this age. (Table 2) Higher scores were also observed in the "satisfaction" domain in patients who underwent surgery after 15 years of age (p<0.05). The β-regression coefficient indicated that for each year of delay from the ideal time of surgery (after age 11), there was an increase of approximately 0.5 points in the SRS-30 "satisfaction" domain. (Table 2)

**Table 2:** SRS30 "Pain domain" and "satisfaction domain" versus age (in years).

	Correlation between age and SRS30 "Pain Domain"							
	Preoperative		6 months		1 year		2 years	
	A	B	A	B	A	B	A	B
Pearson correlation coefficient (*r*)	-0.11	0.11	-0.06	0.29	-0.18	0.28	-0.27	0.19
Total of patients	49	49	49	49	49	49	49	49
p value	0.454	0.449	0.682	0.044	0.22	0.047	0.064	0.194

p: Linear regression analysis. A: Correlation between age and SRS30 "Pain Domain". B: Correlation between age and SRS-30 "Satisfaction Domain".

The correlation between the SRS-30 "function" and "pain" domains with the use of corsets during conservative treatment are shown in Tables 3 and ^4^. It was possible to verify that these patients had worse results in comparison to patients without brace treatment (p=0.005). Furthermore, the use of braces did not influence the other SRS-30 domains after surgical treatment.

**Table 3:** SRS30 "Function domain" versus "use of braces".

Use of braces	SRS30 - "Function Domain"			
	Preoperative	6 months	1 year	2 years
No				
Mean ± SD.	17.9 ± 3.7	25 ± 4.3	25.8 ± 3.9	25.7 ± 4
Median	18.0	25.0	26.0	26.0
Min - Max	11 - 23	14 - 33	20 - 32	20 - 33
Total of patients	33	33	33	33
Yes				
Mean ± SD.	20.1 ± 3.7	27.1 ± 5.1	28.5 ± 3.2	28.6 ± 3.5
Median	21.5	29.0	29.0	29.0
Min - Max	10 - 23	16 - 33	20 - 33	23 - 35
Total of patients	16	16	16	16
p value (time VS use of braces)		p= 0.873		
p value (time)		p <0.001		
p value (use of braces)		p= 0.005		

p: Analysis of variance with repeated measures and a fixed factor.

**Table 4:** SRS30 "Pain domain" versus "use of braces".

Use of braces	SRS30 - "Pain Domain"			
	Preoperative	6 months	1 year	2 years
No				
Mean ± SD.	19.9 ± 3.5	24 ± 4.5	24.8 ± 4.1	25.3 ± 4.1
Median	20.0	25.0	25.0	26.0
Min - Max	13 - 25	10 - 30	16 - 30	16 - 30
Total of patients	33	33	33	33
Yes				
Mean ± SD.	22.4 ± 2.4	25.7 ± 5.3	26.9 ± 2.2	27.1 ± 2.8
Median	22.5	28.0	28.0	28.0
Min - Max	15 - 25	8 - 30	21 - 30	21 - 30
Total of patients	16	16	16	16
p value (Time VS Use of Braces)		p= 0.931		
p value (Time)		p < 0.001		
p value (Use of Braces)		p= 0.038		

p: Analysis of variance with repeated measures and a fixed factor.

There was no correlation between degree of coronal correction of the main thoracic curve and SRS-30 scores. (Table 5) After surgery, there was significant improvement of the five domains of the SRS-30 (p<0.001) and these results were maintained in subsequent evaluations. (Table 6)

**Table 5:** SRS30 total score versus main curve correction.

Curve correction	SRS30 - Total score			
	Preoperative	6 months	1 year	2 years
< 50%				
Mean ± SD.	85 ± 11.4	122.3 ± 3.5	127 ± 6.2	121.3 ± 12
Median	80.0	122.0	125.0	122.0
Min - Max	77 - 98	119 - 126	122 - 134	109 - 133
Total of patients	3	3	3	3
51 - 70%				
Mean ± SD.	82 ± 11.8	120.5 ± 18.4	125.3 ± 12.8	121.4 ± 12.5
Median	82.0	126.0	126.0	119.0
Min - Max	61 - 107	68 - 142	95 - 142	95 - 142
Total of patients	23	23	23	23
>= 71%				
Mean ± SD.	79.3 ± 12	120.4 ± 13.1	122.7 ± 11.3	125.3 ± 12
Median	76.0	120.0	125.0	127.0
Min - Max	60 - 101	93 - 138	97 - 140	96 - 144
Total of patients	23	23	23	23
p value (time VS main curve correction)		p= 0.309		
p value (time)		p < 0.001		
p value (main curve correction)		p= 0.951		

p: Analysis of variance with repeated measures and a fixed factor.

## DISCUSSION

The impact of surgery and perioperative predictive factors remains controversial in AIS treatment. Unlike the present prospective study, most current reports that assessed the quality of life after spine fusion to treat AIS are retrospective studies.^2^ Some authors have used meta-analysis to demonstrate no change in patient quality of life after surgical treatment. However, in the present study we found significant improvement in all functional SRS-30 domains two years after surgery. 

Our results suggest a positive correlation between patient satisfaction and age at the time of surgery. Patients operated after 15 years of age were more satisfied with surgery than younger ones (p<0.05). We believe that individual factors such as education and psychological changes during adolescence may justify such findings. We also observed that age at the time of surgery had an impact on patients' back pain (dorsal and/or lumbar). Patients who received surgery at the end of adolescence had more back pain. This phenomenon was not observed in younger patients. The reasons for these findings are not completely clear; we hypothesize that scoliotic deformities produce asymmetric load distribution on facet joints, which may explain early degeneration of articular cartilage and consequent pain. Unfortunately, this theory is not part of the objective of the present study and was not proven. 

Male patients had higher "mental health" domain scores in comparison to female subjects (p<0.035). This finding was consistent with a previous report that described better results in the "appearance" and "mental health" domains as well as less postoperative pain in males.^11^ In this study, patients of both sexes showed significant improvement regarding the domain "appearance." Other studies compared AIS fusion results and showed no direct association with "mental health" and sex.^12^ ^,^ ^13^

In the present study, braces exerted a negative effect on postoperative back pain, personal satisfaction and overall function (p=0.05). Our findings were consistent with those previously described by Diab et al.^14^ Other studies also have found negative effects regarding the use of braces prior to surgery.^15^ ^,^ ^16^ One reason could be the fact that braced patients were probably less active (in sports and daily activities) than non-braced patients and consequently presented hypotrophy of the trunk stabilization muscles. However, this outcome was not addressed in our study. Future studies about muscle activity in AIS may answer this specific question.

The impact of the amount of correction in functional outcomes of AIS patients is not well established. D'Andrea et al.^4^ and Sanders et al.^16^ reported a weak correlation between curve correction and functional outcomes. Despite the fact that our results do not support a direct correlation between curve correction and functional outcomes, (Table 5) additional studies showed higher rates of complications after major curve corrections (SOSORT study), including iatrogenic trunk imbalance.^17^ In a longitudinal cohort study of 745 patients with AIS who underwent surgical correction, Carreon et al.^18^ observed no statistically significant correlation between the 2-year postoperative SRS satisfaction score and the amount of curve correction (r= 0.07, P= 0.062). However, other studies found high levels of AIS patient satisfaction after deformity correction.^13^ The degree of curve correction after surgery was a significant predictor of function/activity scores, self-image/appearance and satisfaction in 104 Chinese AIS patients.^19^

**Table 6:** SRS30 total score versus time.

SRS30 Domains	SRS30 - Total score			
	Preoperative	6 months	1 year	2 years
Function				
Mean ± SD.	18.6 ± 3.8	25.7 ± 4.6	26.7 ± 3.9	26.7 ± 4
Median	19.0	27.0	27.0	27.0
Min - Max	10 - 23	14 - 33	20 - 33	20 - 35
Total of patients	49	49	49	49
Pain				
Mean ± SD.	20.7 ± 3.4	24.6 ± 4.8	25.5 ± 3.7	25.9 ± 3.8
Median	21.0	26.0	26.0	26.0
Min - Max	13 - 25	8 - 30	16 - 30	16 - 30
Total of patients	49	49	49	49
Appearance				
Mean ± SD.	17.3 ± 4.3	36.3 ± 4.8	36.9 ± 4.5	36.3 ± 4.5
Median	17.0	37.0	37.0	36.0
Min - Max	8 - 28	22 - 45	24 - 45	24 - 45
Total of patients	49	49	49	49
Mental health				
Mean ± SD.	17.5 ± 3.7	20.4 ± 4.2	21.3 ± 3.2	20.9 ± 3.4
Median	17.0	21.0	22.0	21.0
Min - Max	11 - 25	9 - 25	14 - 25	13 - 25
Total of patients	49	49	49	49
Satisfaction				
Mean ± SD.	6.8 ± 2.1	13.7 ± 2.0	13.8 ± 1.9	13.5 ± 1.8
Median	6.0	14.0	15.0	14.0
Min - Max	2 - 10	8 - 15	8 - 15	8 - 15
Total of patients	49	49	49	49
Total				
Mean ± SD.	80.9 ± 11.7	120.6 ± 15.3	124.2 ± 11.7	123.3 ± 12.2
Median	79.0	123.0	125.0	120.0
Min - Max	60 - 107	68 - 142	95 - 142	95 - 144
Total of patients	49	49	49	49

The incidence of complications was low and minor, as previously described.^20^ We experienced 3 wound infections, but only one patient had a deep wound infection and needed reoperation. All patients were treated with oral antibiotics. There was no reported dural perforation, implant loosening, or neurological deficit. We did not find differences among functional outcomes of patients who presented or did not present surgical complications. This may be attributed to the low sociocultural profile of patients included in the present study, who were satisfied even when such complications were present. Moreover, the complications observed were minor and presented satisfactory resolution. 

## CONCLUSIONS

Posterior spine fusion to treat AIS led to improvement in all domains of the SRS-30 questionnaire. Clinical results were influenced by age, sex and the use of braces prior to the surgery. There was no correlation between functional outcomes and the amount of curve correction and the presence of minor perioperative complications.
